# Novel Antibacterial Activity of *Lactococcus Lactis* Subspecies *Lactis* Z_11_ Isolated from Zabady

**Published:** 2013-09

**Authors:** Gamal Enan, Seham Abdel-Shafi, Sahar Ouda, Sally Negm

**Affiliations:** 1 Department of Botany and Microbiology, Faculty of Science, Zagazig University, Egypt;; 2 Department of Plant Research, Nuclear Research Center, Atomic Energy Authority, Egypt

**Keywords:** *Lactococcus lactis* subsp. *lactis* Z_11_, inhibitory activity, bacteriocin, pathogenic bacteria

## Abstract

The purpose of this study was to select and characterize a probiotic bacterium with distinctive antimicrobial activities. In this respect, *Lactococcus lactis* subspecies *lactis* Z_11_ (*L. lactis* Z_11_) isolated from Zabady (Arabian yoghurt) inhibited other strains of lactic acid bacteria and some food-born pathogens including *Listeria monocytogenes, Bacillus cereus *and* staphylococcus aureus. *The inhibitory activity of cell free supernatant (CFS) of *L. lactis* Z_11_ isolated from zabady was lost by proteolytic enzymes, heat resistant. Consequently, the active substance(s) of CFS was characterized as a bacteriocin. This bacteriocin has been shown to consist of protein but has no lipidic or glucidic moieties in its active molecule. Its activity was stable in the pH range 2.0 to 7.0 and was not affected by organic solvents. The *L. lactis Z_11_* bacteriocin was produced in CFS throughout the mide to the late exponential phase of growth of the producer organism and maximum bacteriocin production was obtained at initial pH 6.5 at incubation temperature of about 30°C.

## INTRODUCTION

Lactic acid bacteria are commonly found in foods including fermented meat, vegetables, fruits, beverages and dairy products and also in intestinal, genital and respiratory tracts of men and animals (1). They possessed certain interest, due to their use as probiotic bacteria science they improve the nutritional benefits for health as they have been reported to antagonise pathogenic bacteria in human gut by their antimicrobial metabolites such as bacteriocins, diacetyl, hydrogen peroxide, acetaldehyde, ethanol, organic acids and carbon dioxide (2). They were reported also to produce enzymes such as cellulase, protease and hydroxylase which is involved in conversion of precipitated bile salts and cholesterol derivatives to soluble compounds to be easily excreted by kidney. Therefore, they are an ideal probiotics (3, 4, 22).

Bacteriocins are proteinaceous compounds produced by bacteria that exhibit a bactericidal or bacteriostatic mode of action against sensitive Gram positive and Gram negative bacteria (5, 6, 24, 25). The bacteriocin nisin produced by many strains of *Lactococcus lactis* is used in USA and Europe as food biopreservative (6). Unfortunately, nisin is inactive at pH 7. This clearly suggests that there is a need to continue research in obtaining lactic cultures which could produce antimicrobial compounds at neutral, acidic and alkaline pH values.

The interest in *Lactococcus lactis* has been shown due to (1) its use as a probiotic bacterium and its application as starter culture for yoghurt and cheese industry (7, 21). The present study was undertaken to select and characterize a probiotic bacterium (*Lactococcus lactis* isolated from Arabiian yoghurt) producer of bacteriocin.

## MATERIAL AND METHODS

### Bacterial strains and media

*L. lactis* Z_11_: the producer of inhibitory substance, was isolated from Arabian yoghurt (Zabady) made without starter culture using M17 agar media (Difco). The strain was identified by physiological and biochemical tests (8) and API 50 carbohydrate galleries (Biomerieux, Marcy-1.Etoile, France). To complete the identification of Z_11_ strain DNA was extracted and 16 S r RNA gene was amplified by PCR reaction (9). The PCR products were electrophoresed via agarose gel (9) and were extracted from gel by gene purification kit (Promega). Purified 16 S r RNA gene was sequenced as described previously (9, 10). The 16 S r RNA gene sequence was submitted to gene bank under accession number T1588267 and was compared to the recorded sequences as described previously (12).

The indicator organisms are listed in Table 1. Lactic acid bacteria used in this study were maintained as frozen stocks at -20°C in brain heart infusion (BHI) broth plus 20% glycerol (5), and were propagated in the same media. All other indicator strains were maintained as frozen stocks at -20°C in glass beads (Oxoid) and were propagated in BHI broth (5).

### Preparation of cell free supernatant

*L. lactis* Z_11_ was grown in M17 broth for 14 h at 30°C. Cell-free supernatant was obtained by centrifuging the culture (10000× g for min at 4°C). The pH of the supernatant was adjusted to pH 6.5 with 1 M NaOH and was sterilized by filtration (Amicon, 0.45 μm, Milipore). This pH adjusted, filter sterilized cell-free supernatant was further designated CFS and was used immediately in the experiments (5, 6). *Lactococcus lactis* MG1614 was used as the indicator organism in the whole experiments.

### Antibacterial spectrum of *L. lactis Z_11_*

The agar well diffusion method was used for bioassay of inhibitory activity of CFS (4, 5). A 1% v/v suspension of log-phase cells (2 × 10^3^ CFU/ml) of every indicator strain culture was inoculated in its appropriate soft agar (0.7% agar) and overlaid on the surface of BHI agar plates. After solidification, wells were perforated with a sterile 7 mm cork porer. 50 ml of the CFS were inoculated into the wells and allowed to diffuse into the agar for 4 h at 4°C. The plates were then incubated at 30°C and examined after 12 and 24 h for zones of inhibition (26).

### Estimation of the antibacterial titer of CFS preparation

The quantitative estimation of the antibacterial titer of CFS preparation was performed as described previously (13). One arbitrary unit (AU) was defined as 5 ml of the highest dilution of filtrate yielding a definite zone of growth inhibition on the lawn of indicator organism. The highest dilution was multiplied by 200 (1 ml/5 µl) to obtain the arbitrary units per ml (AU/ml).

### Sensitivity of bacteriocin to enzymes, heat, organic solvents and pH values

To test for enzyme sensitivity, samples of the CFS preparation (1880 AU/ml) were treated with the following filter-sterilized enzymes: pepsin, trypsin, α-chemotrypsin, lipase, amylase (Sigma) at 1 mg/ml final concentration in 10 mM potassium phosphate buffer (pH 6.5). Controls were buffers, CFS without enzymes. Samples and controls were incubated at 37°C for 1 h and tested for remaining antibacterial activity (5, 13, 24, 25, 26).

To test for heat sensitivity, aliquots of CFS (1880AU/ml) were mixed with 10% (v/v) of the organic solvents listed in Table 2. Controls were CFS without organic solvents and 10 mM potassium phosphate buffer (pH 6.5) mixed with organic solvents. Samples and controls were incubated overnight at 60°C to allow solvents to evaporate and tested for remaining antibacterial activity (3).

To test the effect of pH values on the bacteriocin stability , aliquots of CFS (1880 AU/ml) and samples of fresh M17 broth (controls) were adjusted to different pH values listed in Table 3, and incubated for 24 h at 25°C. After setting pH to 6.5 with 10 mM potassium phosphate buffer, samples and controls were tested for remaining antibacterial activity.

To test for heat sensitivity, aliquots of CFS (1880 AU/ml) were heated at 60, 70, 80, 90, 100°C. Every 5 min, 1 ml samples from heat treatments were taken and tested for residual antibacterial activity.

### Effect of incubation temperature and initial pH values on the production of *L. lactis Z_11_* bacteriocin in CFS

To study the influence of the initial pH of the medium and time and temperature during the incubation on the bacteriocin activity of CFS collected from *L. lactis* Z_11_, a series of 500 ml Erlenmeyer flasks, each containing 250 ml M17 broth were adjusted initially with either HCl or NaOH (1M) to various pH values (pH 5.0, 5.5, 6.0, 6.5, 7.0, 7.5 and 8.0) and inoculated with 1% v/v of log phase cells of *L. lactis* Z_11_ at a concentration of about 2 × 10^3^ (CFU/ml). The flasks were then incubated at 25°C, 30°C and 42°C for 96 h. Every 6 h, samples were removed and examined for growth (CFU/ml) and antibacterial activity (AU/ml).

## RESULTS

The Z_11_ strain was Gram positive, catalase negative, and showed coccoid cells. The strain showed the following characteristics: production of DL-isomers of lactic acid from carbohydrates, homofermentation of glucose and fructose, and characteristic sugar fermentation pattern with API 20 (API Streps, Montalieu, Vercieu, France), as described in the manufacturer's instructions. Further identification of Z_11_ strain was carried out by comparison of the sequence of 16 S r RNA gene of DNA with that of Gene Bank and the similarity assignment showed that the Z_11_ strain was classified and identified as belonging to *Lactococcus lactis *subspecies *lactis* and designated *L. lactis* Z_11_.

**Figure 1:**
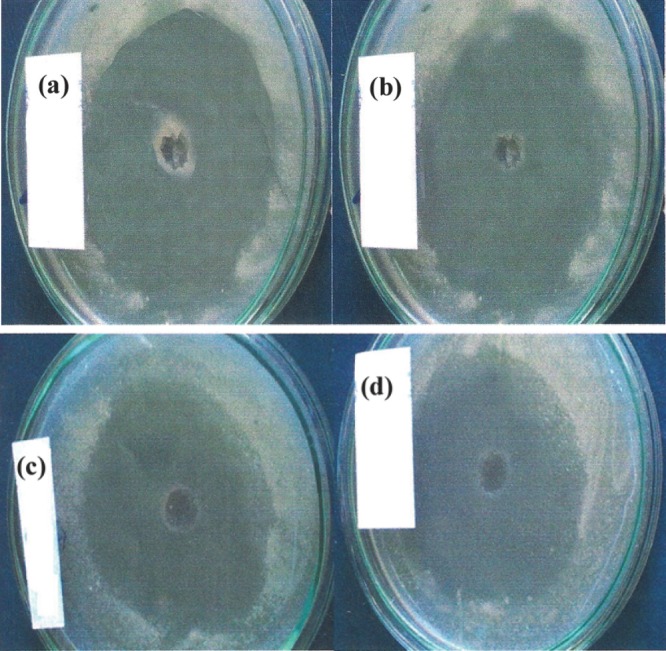
Antibacterial activity of CFS from *L. lactis* Z11 against (a) *Listeria monocytogenes*, (b) *Bacillus cereus*, (c) *Staphylococcus aureus*, (d) *Lactococcus lactis* MG 1614 by the agar well diffusion assay using brain heart infusion agar medium.

In the preliminary screening of lactic acid bacteria cultures for production of inhibitory activity, *L. lactis* Z_11_ was the best strain in production of inhibitory activity and hence, was choosed for further study. CFS preparation were collected from Z_11_ strain and were tested for their inhibitory activity by the agar well diffusion assay against some food-born pathogens. Results are given in Figure 1. The inhibition zones around wells measured ≥30 mm in petri plates containing lawns of indicator organisms viz. *Listeria monocytogenes, Bacillus cereus, Staphylococcus aureus *and* Lactococcus lactis MG1614.* It was necessary to estimate the titer of inhibitory activity against sensitive organisms. Critical dilution assay was followed. Results are given in Table 1. The antibacterial activity showed distinctive titre(s) against Gram positive bacteria ranging from 1020-1880 AU/ml. However, Gram negative bacteria had no response.

**Table 1:** Indicator Strains and their sensitivity to CFS from *L. lactis Z_11_*

Indicator Strain	Source of strains	AU/ml^a^

***Listeria monocytogenes***	LMG 10470	1360
***Bacillus cereus***	LMG 14579	1200
***Stophylococcus aureus***	DSM 1104	1020
***Escherichia coli***	MIR 302	0
***Pseudomonas aeruginosa***	MIR 122	0
***Klebsiella pnemoniae***	MIR 630	0
***Lactococcus lactis***	LIM-MG1614	1880
***Lactobacillus plantarum***	UG1	1620
***Lactobacillus sake***	Lb706	0
***Lactobacillus casei***	LMG 8152	1220
***Lactobacillus alimentarius***	HL2	1440

LMG, Laboratorium voor Mikrobilogie, Gent culture collection, Universiteit Gent, Belgium; DSM, Deutsche Sammlung von Mikroorganisms und Zellkulturen, Gmb II, Braunschweig, Germany, MIR, Mircen Culture Collection, Ain Shams Faculty of Agriculture, Cairo, Egypt; LIM, Laboratory of Industrial Microbiology and Biocatalysis, Faculty of Bioscience Bioengineering, University of Gent, Belgium; UGI, Universiteit Gent -1 (Enan *el al*., 1996); Lb706, a bacteriocin producer strain provided by Dr. Schilinger, karlsruch, Germany; HL; Chr. Hansens Laboratorium, Demark, horsholm, Denmark;

a Titers of the inhibitory substance in CFS as determined by the critical dilution assay.

**Figure 2:**
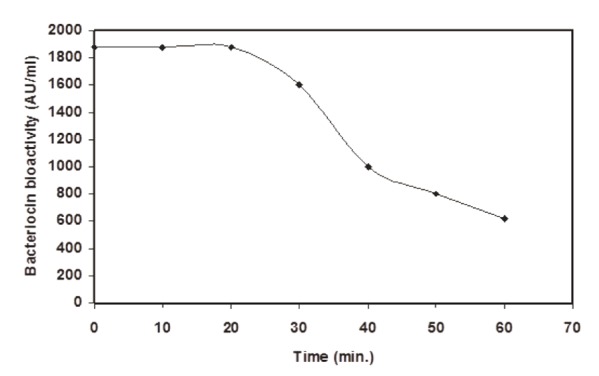
Dynamics of thermal denaturation of *L. lactis Z11* bacteriocin at 100°C for 1 h.

To study the physicochemical properties of CFS of *L. lactis* Z_11_, CFS was collected and was heated for 5 min at 60, 70, 80, 90, 100°C; or treated with many enzymes or organic solvents. Results are given in Table 2. The inhibitory activity was heat resistant, lost by proteolytic enzymes and did not affect by lipase, amylase or organic solvents. This showed that one or more substances of proteinacous nature were responsible for antibacterial activity. It also indicated that an absence of lipidic or carbohydrate moieties and such properties coupled with criteria applied for bacteriocin properties (14). Consequently the inhibitory activity was due to bacteriocin. This bacteriocin was thermo stable and its activity did not affect by heating at 100°C for almost 30 min (Figure 2). The bacteriocin characterized herein was stable at acidic and neutral levels of pH, but partially degraded or was unstable at alkaline levels (Table 3).

**Table 2:** Effect of different treatments on the antibacterial activity of CFS

Treatment	Residual antibacterial activity (Au/ml)

**Temperature**	
100°C for 5 min	1880
90°C for 5 min	1880
80°C for 5 min	1880
70°C for 5 min	1880
60°C for 5 min	1880
**Enzymes**	
Pepsin	0
Trypsin	0
α-Chemdrypsin	0
Lipase	1880
Amylase	1880
**Organic solvents**	
Acetone	1880
Petroleum ether	1880
Methanol	1880
Ethanol	1880
Chloroform	1880

**Table 3:** Effect of different pH values on the stability of the inhibitory substance produced by *L. lactis Z_11_*

pH Value	Bacteriocin activity (AU/ML)

2.0	1880
3.0	1880
4.0	1880
5.0	1880
6.0	1880
7.0	1840
8.0	600
9.0	400
10	400

**Figure 3:**
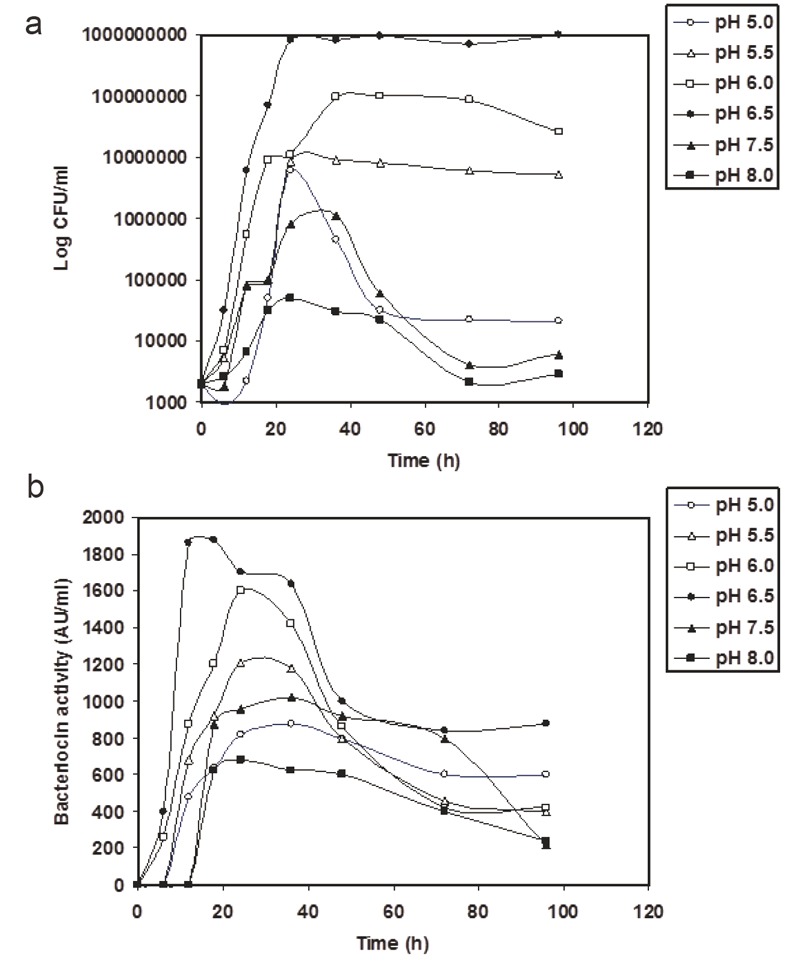
Growth (CFU/ml) (a) and bacteriocin activity (AU/ml) (b) in M17 broth adjusted at intial pH 5(o), 5.5(Δ), 6.0(〈), 6.5(•), 7.5(▲), 8.0(■), inoculated with* L. lactis Z_11_*and incubated at 35°C.

**Figure 4:**
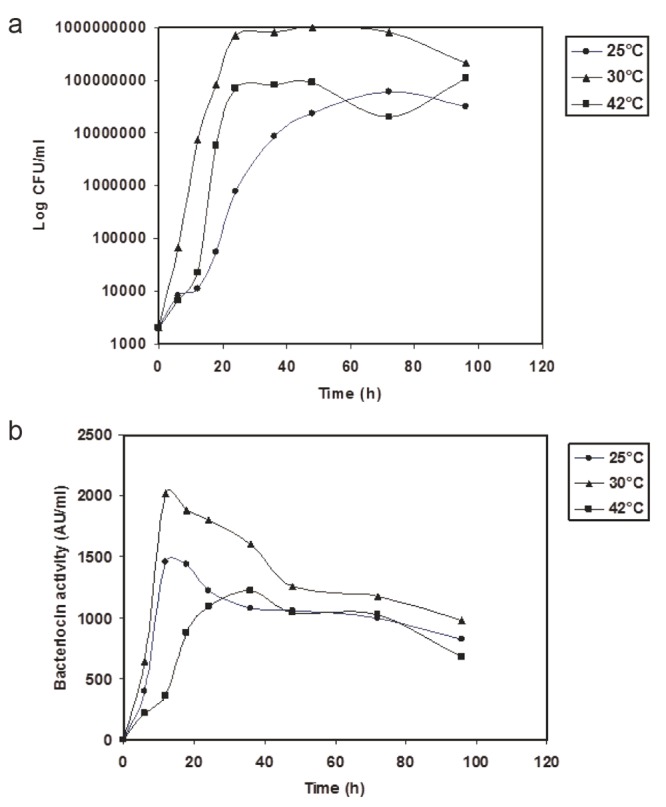
Growth (CFU/ml) (a) and bacteriocin activity (AU/ml) (b) in M17 broth adjusted at intial pH 6.5, inoculated with *L. lactis Z_11_* and incubated at 25°C (•), 30°C (▲) and 42°C (■).

The effect of the initial pH value of the medium and incubation temperature on the production of bacteriocin in CFS by *L. lactis* Z_11_ is shown in Figures 3 and Figure 4. Little amounts of bacteriocin activity and scant values of growth were obtained when *L. lactis* Z_11_ was grown in M 17 broth adjusted initially at pH values 5.0, 7.5 or 8.0. However, the best levels of bacteriocin activity in CFS (1900 AU/ml) were coupled with maximum values of growth after 14 h of incubation at an initial pH 6.5 (Figure 3). In *L. lactis Z_11_* cultures adjusted at this initial pH and incubated at 25°C, 30°C and 42°C (Figure 4), it was shown that the best incubation temperature for growth and bacteriocin production was at 30°C. A maximum bacteriocin production of about 1900 AU/ml was obtained in CFS after 14 h at 30°C when the producer organism was in the mide to the late exponential phase of growth (Figure, 4 a). The growth and bacteriocin production, however, were rather low in CFS at 42°C (Figure 4a). By further incubation to 98 h, the bacteriocin production decreased gradually reaching 980 AU/ml in cultures grown in media adjusted initially at pH 6.5 and incubated at 30°C (Figure 4a, 4b).

## DISCUSSION

The interest in *Lactococcus lactis *has been due to its application as starter culture for yoghurt and soft cheese making as well as its probiotic use (4). *Lactococcus lactis* is used as a probiotic bacterium because it was shown to improve lactose digestion and absorption in lactose intolerant people. It also has more potential and safety in developing vaccine in human (23). In this study, *Lactococcus lactis* subspecies *lactis* Z_11_ (*L. lactis* Z_11_) was isolated from Arabian Yoghurt and identified by recent genetic techniques (10). It was aimed to select a bacteriocin producer strain of *Lactococcus lactis* and could be used as probiotic bacterium by its ability to antaginise pathogens in human gut. Therefore, the inhibitory effect of *L. lactis Z_11_* noticed herein could not be attributed to organic acids or hydrogen peroxide produced by the culture as CFS preparations were not affected when neutralized or treated with catalase. The antibacterial substance produced by *L. lactis Z_11_* was inactivated by proteolytic enzymes and was inhibited to sensitive Gram positive bacteria including some food-borne pathogens and some other lactic acid bacteria. It was almost resistant to heat. Consequently, it has been coupled with most of the definitions of bacteriocins (15). It was, therefore, characterized as a bacteriocin.

The activity of *L. lactis Z_11_* bacteriocin in CFS was not affected by organic solvents and lipase; probably because of the absence of lipid moieties in the active molecule. The same was observed for some bacteriocins produced by* Lactococcus lactis* (6, 16) but differs from both brevicin and pediocin N5P bacteriocins (17). The activity of this bacteriocin was not affected by amylase indicating on absence of glucidic moieties in the bacteriocin molecule. This is similar to other bacteriocins produced by *Lactococcus lactis* (6). The bacteriocin employed herein was stable at acidic and neutral pH values (from pH 2 to pH 7) and this is promised result since the producer organism: *L. lactis Z_11_* grew well in acidic yoghurt and this could give wider application of *L. lactis Z_11_* in dairy industry at acidic and neutral pH values. This differs from other bacteriocins produced by *Lactococcus lactis* which are unstable at neutral pH value (18), but similar to bacteriocin S 50 produced by* Lactococcus lactis* subspecies *diacetyllactis* which is active in the pH range 2 to 11(19).

The production of *L. lactis Z_11_* bacteriocin in CFS was optimal at an initial pH 6.5 and incubation temperature of about 30°C when the producer organism was in the mide to the late exponential phase of growth with a distinctive decline thereafter. Similar results in bacteriocin activity have been reported for other bacteriocins (15, 19). The decrease in the bacteriocin activity at latter stages of growth may be due to adsorption of the bacteriocin to live and dead cells of the producer organism (15, 16, 26).

In view of bacteriocins produced by *Lactococcus lactis , L. lactis Z_11_* bacteriocin differs from the bacteriocin nisin or some nisin-like bacteriocins which are active at acidic pH values only but not at pH 7 (6, 20), but similar to other bacteriocins in their pH stability and thermostability such as bacteriocin S 50 produced by *Lactococcus lactis subsp. diacetylactis* (19). Generally, comparison of different bacteriocins based upon biological characters is elusive as it is strongly dependent on the variability of strains used and culture condition. Further work will be necessary to find whether the antibacterial activity noticed herein is due to one or more substances and to purify this bacteriocin and analyze its amino acid sequence, molecular mass and its actual classification.
